# The Vitamin A Derivative All-Trans Retinoic Acid Repairs Amyloid-*β*-Induced Double-Strand Breaks in Neural Cells and in the Murine Neocortex

**DOI:** 10.1155/2016/3707406

**Published:** 2016-01-03

**Authors:** Emmanuelle Gruz-Gibelli, Natacha Chessel, Clélia Allioux, Pascale Marin, Françoise Piotton, Geneviève Leuba, François R. Herrmann, Armand Savioz

**Affiliations:** ^1^Department of Psychiatry, University Hospital Geneva, Chêne-Bourg, 1225 Geneva, Switzerland; ^2^Center for Psychiatric Neuroscience, Department of Psychiatry, CHUV, 1008 Lausanne-Prilly, Switzerland; ^3^Department of Internal Medicine, Rehabilitation and Geriatrics, University Hospital of Geneva and University of Geneva, Thônex, 1226 Geneva, Switzerland; ^4^Geneva Neuroscience Center, Geneva University, 1211 Geneva, Switzerland

## Abstract

The amyloid-*β* peptide or A*β* is the key player in the amyloid-cascade hypothesis of Alzheimer's disease. A*β* appears to trigger cell death but also production of double-strand breaks (DSBs) in aging and Alzheimer's disease. All-trans retinoic acid (RA), a derivative of vitamin A, was already known for its neuroprotective effects against the amyloid cascade. It diminishes, for instance, the production of A*β* peptides and their oligomerisation. In the present work we investigated the possible implication of RA receptor (RAR) in repair of A*β*-induced DSBs. We demonstrated that RA, as well as RAR agonist Am80, but not AGN 193109 antagonist, repair A*β*-induced DSBs in SH-SY5Y cells and an astrocytic cell line as well as in the murine cortical tissue of young and aged mice. The nonhomologous end joining pathway and the Ataxia Telangiectasia Mutated kinase were shown to be involved in RA-mediated DSBs repair in the SH-SY5Y cells. Our data suggest that RA, besides increasing cell viability in the cortex of young and even of aged mice, might also result in targeted DNA repair of genes important for cell or synaptic maintenance. This phenomenon would remain functional up to a point when A*β* increase and RA decrease probably lead to a pathological state.

## 1. Introduction

DNA damage, such as DNA single- or double-strand breaks (DSBs), is known to occur in aging [1, 2] as well as in Alzheimer's disease (AD) [2–6]. Recently DSBs were shown to be produced by A*β*  or amyloid-*β* peptides [7, 8] through oxidative stress [9]. Furthermore, A*β* peptides not only increase neuronal vulnerability (e.g., apoptosis) in DNA-dependent Protein Kinase (DNA-PK) deficient mice, a key enzyme of the Nonhomologous End Joining (NHEJ) pathway involved in DSBs repair [10], but also reduce the activity of this enzyme [11, 12]. Thus, extracellular A*β* peptides trigger DSBs production and impair DSBs repair.

However, if numerous factors are contributing to the formation or increased levels of A*β* peptide, such as mainly age, the* apoE4* allele, cholesterol rich food, or glucocorticoid stress hormone dexamethasone, others factors, such as the* apoE2* allele and the growth factor BDNF, are neuroprotective [13, 14] or participate in “adaptive cellular responses” [15]. Among them, some even diminishes DNA damage. This is the case of glutamine that reduces etoposide-induced damage [16] and of NAD that attenuates A*β*-induced DNA damage [8].

In this study we were particularly interested in all-trans retinoic acid (RA) [17–20], a derivative of vitamin A [21]. RA is known to be involved in development, neuronal differentiation [22], spine formation through the RAR*α* receptor [23], cell growth arrest in anticancer therapy [24], and memory decline in aging [25–27]. The neuroprotective role of vitamin A and RA in relation to AD and to the *β*-amyloid cascade—not to DNA DSBs—has intensively been studied, for example, [28]. Firstly, RA was shown to increase, via its RAR*α* receptor, the expression of the major *α*-secretase, ADAM10 (A Disintegrin and Metalloproteinase domain-containing protein 10), diminishing the production of A*β* peptides [29]. This effect is mediated by RA-responsive elements upstream of the ADAM10 coding region [30, 31]. RA can also inhibit the *γ*-secretase activity through activation of the Extracellular Signal Regulated Kinase, ERK1/2 [32]. Secondly, vitamin A and its derivatives appear to inhibit A*β* oligomerisation* in vitro*, A*β* deposition, and tau phosphorylation in AD mouse models [33]. Thirdly, RAR*α* signaling removes A*β* plaques and induces A*β* oligomers clearance via Neprilysin and Insulin Degrading Enzyme [34]. On the contrary, A*β* is increased in the cerebral vasculature while RAR*α* is decreased in the neocortex of rats maintained on a 1-year retinoid-deficient diet [35]. Finally, following a RA treatment in APP/PS1 double-transgenic mice, A*β* deposits, AICDs (the Amyloid precursor protein Internal C-terminal Domains), tau phosphorylation, and glial response were decreased, whereas spatial learning was improved [36].

RAR are major players in the neuroprotective effects of RA. RA by binding to them allows the formation of RAR/RXR heterodimers and the replacement of corepressors, such as HDAC (histone deacetylase), by coactivators, such as CBP (CREB-binding protein). The histone acetyltransferase activity of CBP [37] and the down-regulation of DNA methyltransferases [24] result in RA-dependent transcription. Indeed, RA hypomethylates promoters, such as the one of RAR*β*2 or of methyltransferases, altering gene transcription [24].

According to these data, we wondered if, as a consequence of local chromosome relaxation, DNA repair proteins [2], such as the catalytic subunit of DNA-PK and Ataxia Telangiectasia Mutated kinase (ATM), could be recruited at chromosome sites of RA-dependent gene expression and if DSBs could be repaired at these sites. We have investigated whether RA and its receptor (RAR) might be involved, not in prevention of A*β* synthesis, A*β* oligomerisation, and plaques removal, as already shown, but in repair of A*β*-induced DSBs. We showed—to our knowledge for the first time—that this is indeed the case by studying neuronal and astrocytic cell lines as well as neocortical tissue from young and old mice. Furthermore, we examined the repair mechanisms involved and the consequences on cell viability, in the search for effective early AD therapies.

## 2. Materials and Methods

### 2.1. Mice, Tissue Samples, and Dissections

C57BL/6J male mice (Janvier, Le Genest-St-Isle, France) were sacrificed at 4 months (young adults) and at 16 months (aged adults). Brains were quickly removed and cortices dissected under a binocular microscope, weighted, and homogenized (about 5 sec with a Polytron apparatus, VWR International) in neurobasal medium (Gibco Life Technologies). The homogenized tissue was immediately treated as described below. Animals were handled in accordance with Federal Swiss Veterinary regulations and approval.

### 2.2. Culture of Neuronal SH-SY5Y Cells and of Astrocytic DI TNC_1_ Cells

Human SH-SY5Y cells (European Collection of Animal Cell Culture, UK) were routinely grown in a 37°C incubator containing 5% CO_2_/95% humidified air in RPMI-1640 (Gibco Life Technologies) with 10% FCS (Bioconcept, Switzerland), 2 mM L-glutamine (Gibco Life Technologies), 100 IU/mL penicillin G, and 100 *μ*g/mL streptomycin (Invitrogen). Cells were halved every 5 to 6 days. For this purpose, the confluent cells were released in DPBS (150 mM NaCl, 3 mM KCl, 1.5 mM KH_2_PO_4_, 7.9 mM Na_2_HPO_4_·2H_2_O, and 0.1 mM EDTA; pH 7.4), centrifuged at 1000 rpm during 5 min, and resuspended in RPMI-1640 and 10% FCS at the desired dilution.

The DI TNC_1_ cell line [38, 39] was grown in DMEM medium (Sigma) supplemented with 10% FCS in a 37°C incubator containing 5% CO_2_/95% humidified air. Confluent cells were released in trypsin solution (0.25%), centrifuged at 1100 rpm during 5 min, and resuspended in DMEM with 10% FCS at the desired dilution.

Cells were photographed with an inverted phase contrast microscope (ZEISS Telaval 31) using a photo camera (Leica DFC 490) with the FireCam analyst software (Leica).

### 2.3. Tissue and Cell Treatments

For the cell treatments, two days before experiment, the medium with 10% FCS was replaced by the medium with 1% FCS. The cultured cells and the homogenized cortical tissue (see before) were treated or not with 20 *μ*M monomeric A*β*_1–42_ peptides (Enzo Life Sciences), during the first 30 min, then with or without 5 *μ*M RA (Sigma-Aldrich) for 30 more min, resulting in four combinations of 2 × 30 min treatments (Ø-Ø; A*β*_1–42_-Ø; Ø-RA; A*β*_1–42_-RA). Other treatments were carried out with 20 *μ*M A*β*_42–1_, 20 *μ*M A*β*_1–40_ (Enzo Life Sciences), 10 *μ*M RAR*α*/*β* agonist Am80 (Santa Cruz Biotechnology), 1–50 *μ*M RAR*α*/*β*/*γ* antagonist AGN 193109 (Labforce), 50 *μ*M caspase-3 inhibitor z-VAD-FMK (Santa Cruz Biotechnology) [40], 3 *μ*M HDAC inhibitor trichostatin A (TSA; Sigma) [41], 10 *μ*M inhibitor of Ataxia Telangiectasia Mutated kinase or ATM KU 55933 (KU; Labforce), and 150 *μ*M inhibitor II of the catalytic subunit of DNA-PK NU 7026 (NU; Calbiochem), in combination with RA and/or A*β*_1–42_ for 30 min or 24 h. All treatments were carried out in triplicate in a 37°C incubator containing 5% CO_2_ and 95% humidified air.

### 2.4. Neutral Single-Cell Gel Electrophoresis (Comet Assay)

DSBs were measured in SH-SY5Y cells, in DI TNC_1_ cells, and in the homogenized cortical tissue using Trevigen Comet AssayTM kit (AMS Biotechnology, UK) with the following modifications. After treatment, cells were resuspended in ice-cold Ca^2+^ and Mg^2+^ free PBS to a concentration of 1.0 to 1.5 × 10^5^ cells/mL. The same number of cells from the dissociated cortical tissue was resuspended in the same solution with the addition of 20 mM EDTA and processed as for the cell cultures. An aliquot of 50 *μ*L cells was added to 500 *μ*L of 1% molten low-melting agarose (Seaplaque, FMC BioProduct, USA) kept at 42°C. Fifty microliters was immediately spread on a comet slide (AMS Biotechnology), which was incubated at 4°C in the dark for 10 min to accelerate agarose gelling and then transferred to prechilled lysis solution (AMS Biotechnology) for 60 min at 4°C. Subsequently, the slide was incubated in Neutral Electrophoresis Buffer (500 mM Tris base, 1.5 M sodium acetate, pH 9.0) for 30 min at 4°C. DSBs were separated by electrophoresis at 26 V for 45 min. Then, the slides were immersed in 70% ethanol at room temperature for 30 min and air-dried. DNA was stained 10 min at room temperature with 100 *μ*L SYBR Green I dye (Gibco Life Technologies) diluted 1 : 1000 in water and then rinsed with distilled water. Comets of at least 30 cells per treatments were immediately photographed using an Olympus digital camera attached to an Olympus BX51 epifluorescence microscope (Axio vision rel. 4.6).

### 2.5. Analysis of DSBs on Agarose Gel Electrophoresis

To visualize the average tail length of a treated cell population, 10 *μ*L of SH-SY5Y cells (1 × 10^5^ cells/mL) was mixed with 5 *μ*L lysis buffer (AMS Biotechnology) for 5 min at 4°C and then 1.5 *μ*L Tris EDTA 10x was added. The whole sample was loaded into a slot of 1% agarose MP gel (Roche Life Science). A 1 kb ladder (Invitrogen) was used as a size marker. Electrophoresis was run for 40 min at 66 mV. The agarose gel was stained by ethidium bromide (10 mg/mL) and analyzed with a GS-700 imaging densitometer (Bio-Rad). Signal intensities were measured with the molecular analyst software program (Bio-Rad).

### 2.6. Immunocytochemistry

Cortices of 4- (*n* = 3) and 16-month-old C57BL/6J male mice (*n* = 3) were mechanically dissociated and fixed for 30 min at room temperature in 4% paraformaldehyde in PBS on coverslips pretreated with 100% alcohol. After rinsing for 3 × 5 min with PBS, cells were incubated for 1 h30 with the primary antibody diluted in PBS. The mouse monoclonal anti-bIII-tubulin antibody (Sigma), diluted 1 : 1000 in PBS, and the mouse monoclonal anti-glial fibrillary acidic protein antibody (GFAP, Sigma), diluted 1 : 500, were used. After rinsing for 5 min with PBS, coverslips were incubated for 1 h at room temperature with the secondary anti-mouse IgG antibody coupled to AlexaFluor 488 (Molecular probes/Invitrogen), diluted 1 : 1000, in presence of Dapi (1.0 *μ*g/mL, Sigma) and rinsed for 3 × 5 min with PBS, and then mounted on glass slides fixed with Fluorsave (Calbiochem).

Nine to twelve pictures of immunostained cells, corresponding to 34 to 69 cells, were taken for each mouse using a Zeiss Axioskop 2 plus microscope (Carl Zeiss, Feldbach, Switzerland) equipped with epifluorescence and were digitalized with an Axiocam camera.

### 2.7. Cell Viability Assay

For assessing cell viability of SH-SY5Y cells and DI TNC_1_ cells, 20000 cells/well were grown in 1 mL RPMI-1640 with 10% FCS for 6–8 days and, two days before the experiment, the medium was replaced by RPMI-1640 with 1% FCS. For the cortical tissue, 1 mg freshly homogenized tissue was resuspended in 1 mL of the same medium. The cells were treated or not during 30 min with 5 *μ*M RA and/or 20 *μ*M A*β*_1–42_ monomers or 3.3 *μ*L digitonin (CellTiter-Glo Luminescent Cell Viability Assay, Promega) used as a positive control to decrease cell viability. 100 *μ*L of cell suspension was shaken for 2 min with 100 *μ*L of CellTiter-Glo Reagent prepared according to the manufacturer (CellTiter-Glo Luminescent Cell Viability Assay, Promega) to induce cell lysis and to release the cell ATP content, as indicator of metabolic activity. After 10 min incubation at room temperature, luminescence (in relative light units) was recorded with a luminometer (GloMax 20/20, Promega). Medium without cells or tissue samples resulted in background luminescence.

### 2.8. Caspase-Glo

For assessing caspase-3 and caspase-7 activation, SH-SY5Y cells were grown in 1 mL RPMI-1640 with 10% FCS. Two days before the experiment, the medium was replaced by RPMI-1640 with 1% FCS. Cells were treated or not during 30 min or 24 h with 20 *μ*M A*β*_1–42_, 50 *μ*M z-VAD (in DMSO, Merk), or 100 nM staurosporine (Sigma-Aldrich). 100 *μ*L of cell suspension was mixed for 2 min with 100 *μ*L of Caspase-Glo 3/7 Reagent prepared according to the manufacturer (Caspase-Glo 3/7 Assay, Promega) to induce cell lysis and cleavage of a luminogenic substrate of caspase-3/caspase-7. After 1 h30 incubation at room temperature, luminescence was recorded with a luminometer (GloMax 20/20, Promega). A control without cells was also used.

### 2.9. Statistical Analysis

For the comet assay, values of mean comet tail length were compared for each condition by a one-way analysis of variance (ANOVA) to establish the effects of various treatments. When overall statistically significant differences in treatments effect were obtained by ANOVA, comparisons of means among subgroups were made after Bonferroni corrections. In parallel, the nonparametric Kruskal-Wallis test was used to compare the shape of comet tail distribution also with Bonferroni corrections to compare subgroups. The Kruskal-Wallis analysis is particularly suitable when the number of measures is small (less than 15 per group) or when the distribution is not Gaussian (asymmetric box plot). Level of significance is *P* < 0.05. Analyses were carried out with the Stata 13.1 software (Stat Corp., TX, USA, 2013).

## 3. Results

### 3.1. Retinoic Acid Repairs A*β*-Induced DSBs in SH-SY5Y and DI TNC_1_ Cells

**Figure 1:**
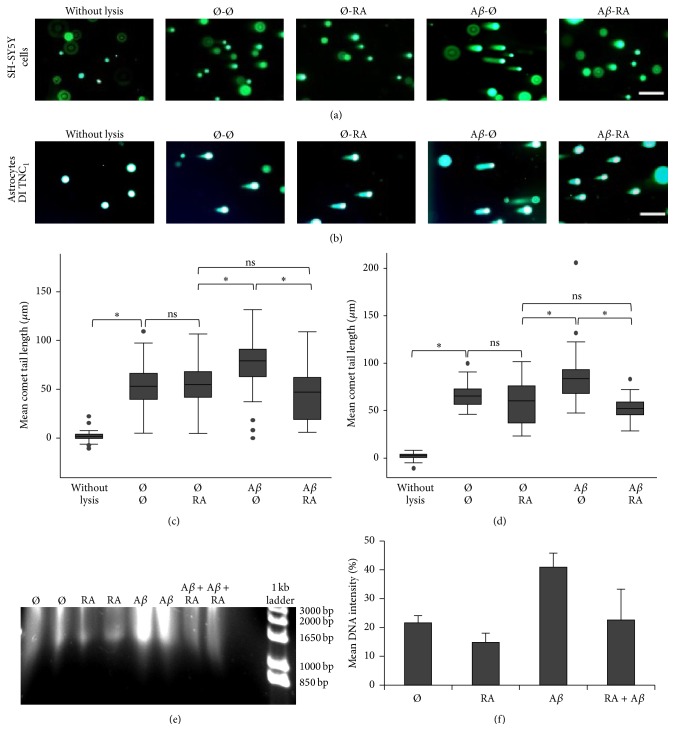
RA repairs A*β*-induced DSBs in SH-SY5Y and astrocytic DI TNC_1_ cells. (a) Representative pictures of comets with various tail lengths of SH-SY5Y cells or of (b) DI TNC_1_ cells following 30 min RA (5 *μ*M) and/or 30 min A*β* (20 *μ*M) treatments. Ø = without treatment for 30 min; scale bar: 200 *μ*m. (c) Box plots of mean comet tail lengths of SH-SY5Y cells (number of cells measured: 31 < *n* < 53) and of (d) DI TNC_1_ cells (31 < *n* < 36). ANOVA with Bonferroni correction: ^*∗*^ *P* < 0.05; ns = not significant. (e) Agarose gel electrophoresis showing comet tails of about 1 × 10^3^ lysed SH-SY5Y cells per well treated with RA (5 *μ*M) and/or A*β* (20 *μ*M). Each treatment was carried out twice. A 1 kb ladder (Invitrogen) was used. (f) Graphical representation of mean optical DNA intensities for each duplicated treatment in (e). It shows that RA decreases the amount of A*β*-induced DSBs and corroborates Fig. (c). The DNA intensities were measured for 1 kb to about 20 kb DNA.

To demonstrate that RA can repair A*β*-induced DSBs, SH-SY5H cells and astrocytic DI TNC_1_ cells were treated with A*β*_1–42_ for half an hour before the addition or not of RA for also 30 min. The presence of RA resulted in shorter tail lengths comparable to untreated lysed cells (Figures 1(a) and 1(b)). The mean comet tail length was significantly higher in A*β* treated SH-SY5Y cells as well as DI TNC_1_ cells compared to all other treatments (Figures 1(c) and 1(d)). These results were corroborated by an independent experiment showing on an agarose gel comet tails starting from their cell nuclei loaded into the gel's slots. Short DNA fragments—between about 0.85 kb and 3.0 kb—were generated more frequently when A*β* was present and were reduced in number in presence of RA (Figure 1(e)). Apoptotic fragments of *n*  × 180 bp could not be detected, suggesting that apoptosis was not activated under these 30 min treatments. Quantification of signals intensities up to the gel's slots revealed that DSBs were almost halved when RA was added after A*β* (Figure 1(f)).

### 3.2. Retinoic Acid Repairs A*β*_1–42_-Induced DSBs in a Concentration-Dependent Manner via RAR*α*/*β* in SH-SY5Y Cells

**Figure 2:**
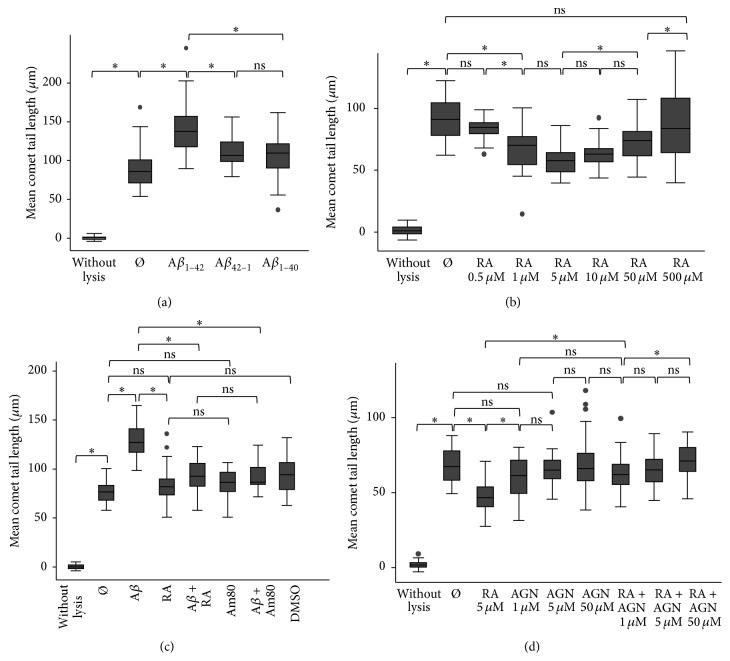
Control experiments for the effects of A*β* and RA on mean comet tail lengths in SH-SY5Y cells. (a) Box plot of mean comet tail lengths according to 30 min treatments with A*β*_1–42_ and control peptides A*β*_42–1_ or A*β*_1–40_ (20 *μ*M A*β*; number of cells measured: 33 < *n* < 48). (b) Box plot of mean comet tail lengths depending on 30 min treatments of various RA concentrations (25 < *n* < 35). (c) Box plot of mean comet tail lengths according to 30 min treatments with 20 *μ*M A*β*_1–42_, 5 *μ*M RA, 10 *μ*M RAR*α*/*β* agonist Am80, and its solvent DMSO (1% v/v), demonstrating a similar specific effect of RA and of Am80 in SH-SY5Y cells (26 < *n* < 41). (d) Box plot of mean comet tail lengths according to the RAR*α*/*β*/*γ* antagonist AGN 193109 (1–50 *μ*M AGN) in presence or absence of 5 *μ*M RA (28 < *n* < 49). The reduced mean tail length due to RA is abolished in presence of AGN. ANOVA with Bonferroni correction: ^*∗*^ *P* < 0.05; ns = not significant; Ø = without treatment for 30 min.

We demonstrated that DSBs are induced specifically by the A*β*_1–42_ peptide and not by the nonpathological A*β*_1–40_ form or a control peptide with the reverse sequence A*β*_42–1_, all treatments being carried out for 30 min with 20 *μ*M A*β* peptides (Figure 2(a)). A similar result was obtained with DI TNC_1_ cells. Furthermore, a dose-response curve (Figure 2(b)) showed that RA repairs DSBs most efficiently at concentrations between 1 *μ*M and 50 *μ*M, with a peak of maximal efficiency at 5 *μ*M, whereas it had no effect at 0.5 *μ*M or 500 *μ*M. Using a cell viability assay, we observed that a treatment with 0.5 *μ*M RA (101.3 ± 15.3%) and 1 *μ*M RA (103.8 ± 8.6) resulted in a similar viability compared to that of the untreated cells (100 ± 10%). However, the viability increased at 5 *μ*M RA (135.8 ± 30.8%), 10 *μ*M RA (152 ± 12.7%), and 50 *μ*M RA (126 ± 30.2) but dropped at 500 *μ*M RA (27.1 ± 2.1) (*n* = 3).

Furthermore, we demonstrated in SH-SY5Y cells that the RAR*α*/*β* agonist Am80 used at a concentration of 10 *μ*M had the same effect compared to RA (Figure 2(c)). Both significantly reduced A*β*-induced DSBs. Moreover, no significant difference was observed between Am80 and RA suggesting that in SH-SY5Y cells the RAR*α*/*β* receptor was sufficient to mediate all DSBs repair activity, thus excluding the involvement of other potential receptors, such as PPAR*β*/*δ* [42]. Finally, the addition of 1 *μ*M RAR*α*/*β*/*γ* antagonist AGN 193109 to 5 *μ*M RA significantly impaired the diminution of DSBs due to the RA treatment. This effect was even increased with a higher concentration of AGN (50 *μ*M compared to 1 *μ*M), clearly demonstrating that the binding of RA to its RAR is needed to repair DSBs (Figure 2(d)).

### 3.3. Retinoic Acid Repairs A*β*-Induced DSBs in the Neocortex of Young and Aged C57BL/6J Mice

**Figure 3:**
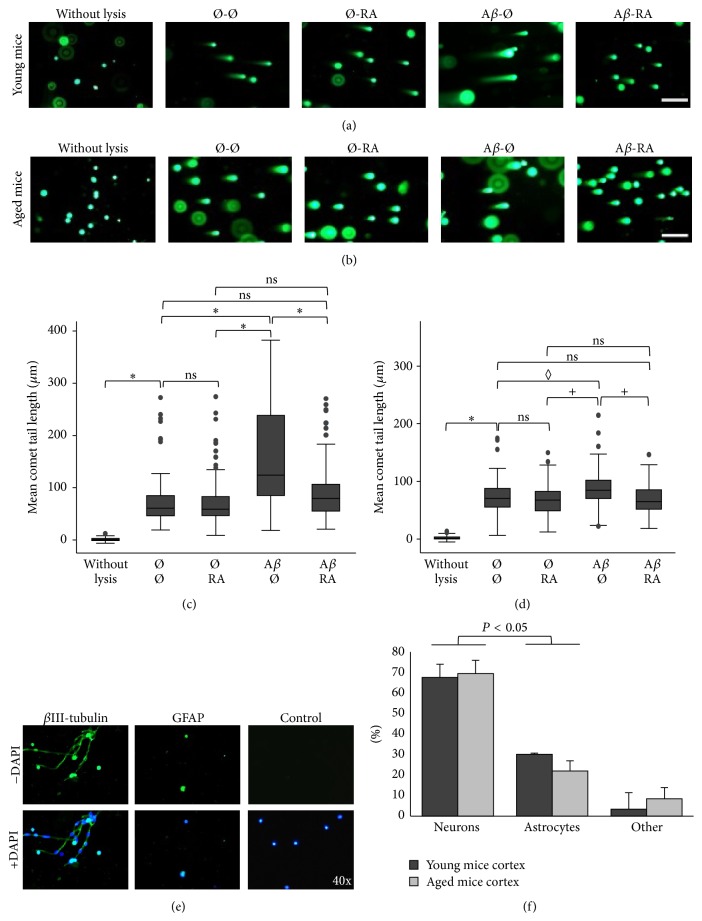
RA repairs A*β*-induced DSBs in the neocortex of C57BL/6J mice. (a) Representative pictures of comets with various tail lengths of cortical cells originating from young (4 months; *n* = 3 mice) or (b) aged (16 months; *n* = 3) mice following 30 min RA (5 *μ*M) and/or 30 min A*β* (20 *μ*M)* in vitro* treatments. Ø = without treatment for 30 min; scale bar: 200 *μ*m. (c) Box plots of mean comet tail lengths of 3 young (number of cells measured: 31 < *n* < 56) and of (d) 3 aged mice (30 < *n* < 51). Statistical analyses (ANOVA with Bonferroni correction) revealed significant differences in all 3 young mice (^*∗*^ *P* < 0.05), whereas only 2 out of 3 (^+^ *P* < 0.05) or 1 out of 3 aged mice (^◊^ *P* < 0.05) reached statistical significance; ns = not significant. (e) Immunofluorescent pictures of cortical cells of young mice stained with anti-*β*III-tubulin to mark neurons, anti-GFAP to label astrocytes, and DAPI as a nuclear marker. A similar study war carried out with cortical cells of aged mice. (f) Proportions of neurons, astrocytes, and other cell types in the cortex of young (*n* = 3) and of aged mice (*n* = 3). Number of cells analyzed for each mouse: 33 < *n* < 70. *P* < 0.05: comparison between neurons and astrocytes.

RA added half an hour after the A*β*_1–42_ treatment can also repair DSBs in cells originating from the murine cortex of young (*n* = 3) and aged C57BL/6J mice (*n* = 3). The presence of RA resulted in shorter tail lengths comparable to untreated lysed cells and cells treated with RA alone (Figures 3(a) and 3(b)). The mean comet tail length was significantly higher in A*β* treated cortical cells compared to all other conditions in the young as well as in the aged mice (Figures 3(c) and 3(d)). However, the difference in mean comet tail lengths between the A*β* treatment and the other conditions was less important in the aged compared to the young mice possibly due to a decreased metabolism. Indeed, the difference between the A*β* and the A*β* + RA treatment was statistically different in all 3 young mice, but only in 2 aged mice out of 3. We further showed by immunocytochemistry (Figure 3(e)) that the cells analyzed in the comet assay consist in neurons (68% for pooled age groups) as well as astrocytes (26%) and that the ratio of astrocytes/neurons (0.38) was similar to that reported in the literature [43] (Figure 3(f)).

### 3.4. Histone Deacetylase Inhibitor Trichostatin A and/or Retinoic Acid Induce DSBs Repair through DNA-PK and ATM in SH-SY5Y Cells

**Figure 4:**
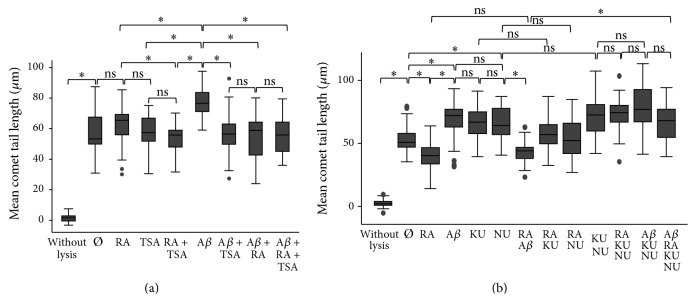
Repair pathways of A*β*-induced DSBs. (a) Box plot of mean comet tail lengths of SH-SY5Y cells (number of cells measured: 31 < *n* < 47) after 30 min treatments with RA (5 *μ*M), the HDAC inhibitor TSA (3 *μ*M), and A*β* (20 *μ*M) demonstrating the involvement of HDAC. (b) Box plot of mean comet tail lengths of SH-SY5Y cells (20 < *n* < 42) after 30 min treatments with RA (5 *μ*M), A*β* (20 *μ*M), KU 55933 (ATM inhibitor, 10 *μ*M), and NU 7026 (DNA-PK inhibitor, 150 *μ*M) demonstrating the involvement of ATM and of DNA-PK. ANOVA with Bonferroni correction: ^*∗*^ *P* < 0.05; ns = not significant; Ø = without treatment for 30 min.

To ascertain the mode of action of RA via its receptor RAR, SH-SY5Y cells were treated with histone deacetylase (HDAC) inhibitor trichostatin A (TSA). We observed that TSA decreased mean tail length produced by A*β* as efficiently as RA (Figure 4(a)). Moreover, DSBs were significantly decreased in cells treated with TSA + RA compared to RA treated cell, suggesting a possible synergetic effect of both chemicals as they act at different sites of the same receptor complex [37].

Moreover, the inhibition of the RA repair activity was shown to be mediated both by ATM using ATM inhibitor KU 55933 and by DNA-PK using DNA-PK inhibitor NU 7026 (Figure 4(b)). The ATM inhibitor induced as many DSBs as the DNA-PK inhibitor or the A*β* treatment. In presence of RA, inhibition of both pathways, but not of only one, produced a similar amount of DSBs as the A*β* treatment. Thus, both ATM and DNA-PK protein kinases need to be present, so that RA is able to repair DSBs in SH-SY5Y cells.

### 3.5. Effects of A*β* on Apoptosis and of RA on Cell Viability

**Figure 5:**
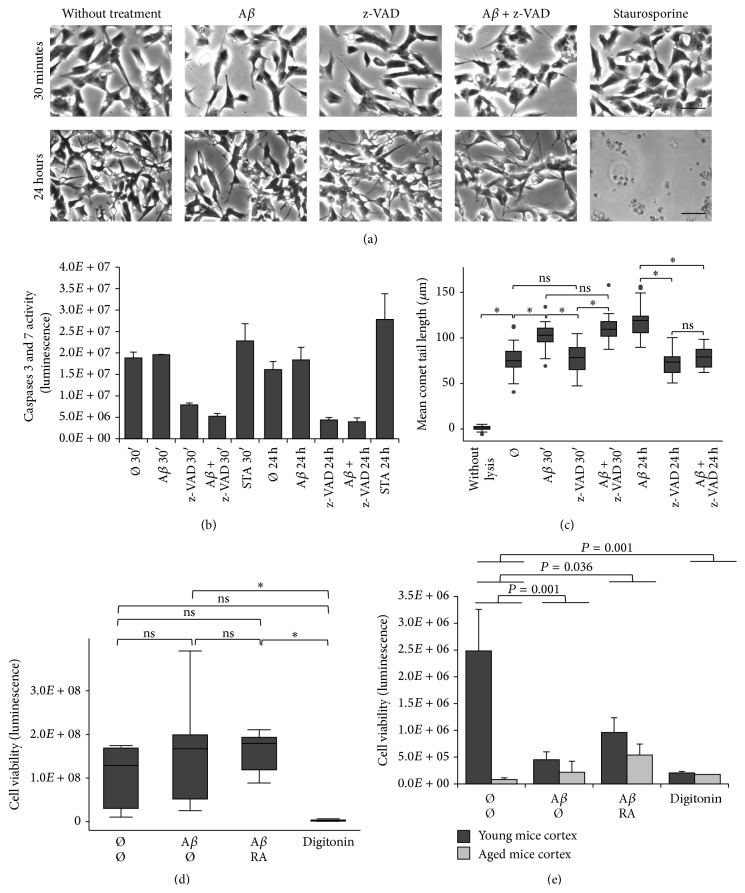
A*β* effects on the apoptotic cascade, on DSBs, and on cell viability in presence of the caspase-3 inhibitor z-VAD or RA. (a) Pictures of phase contrast microscopy with SH-SY5Y cells treated with 20 *μ*M A*β* and/or 50 *μ*M z-VAD did not show cell death, whereas treatment with 100 nM staurosporine resulted in apoptotic cell death after 24 h, not after 30 min. Scale bar = 200 *μ*m. (b) Measures of caspase-3/caspase-7 (CaspaseGlo assay) after 30 min and 24 h treatment with A*β* and/or z-VAD or staurosporine (STA). Caspases were activated in the control cell cultures (Ø = without treatment) and in presence of A*β* after 30 min and 24 h but were not able to induce cell death. A nonsignificant increase of caspases between 30 min and 24 h resulted in cell death (a). (c) Box plot of mean comet tail lengths after treatments with A*β* and/or z-VAD shows that A*β*-induced DSBs in SH-SY5Y are not related to apoptotic DSBs after 30 min, whereas they are related to apoptotic DSBs at 24 h (number of cells measured: 27 < *n* < 38). (d) Measures of viability (Glomax assay) of astrocytic DI TNC_1_ cells treated for 30 min with RA (5 *μ*M) and/or A*β* (20 *μ*M) or digitonin (3.3 mM) (*n* = 9). A similar result was observed with SH-SY5Y cells, that is, no alteration of viability despite RA and/or A*β* treatments. (e) Measures of viability (Glomax assay) of cells from cortical tissue of young (dark gray; 4 months; *n* = 3) and aged mice (light gray; 16 months; *n* = 3) after treatments with RA and/or A*β* or digitonin during 30 min. Viability is less significantly decreased in presence of RA. ANOVA with Bonferroni correction: ^*∗*^ *P* < 0.05; ns = not significant; Ø = without treatment for 30 min.

SH-SY5Y cells were treated with A*β* and/or caspase-3 inhibitor z-VAD. Staurosporine was used as positive control for apoptotic cell death. Only this last compound resulted in cell death after 24 h, but not after 30 min treatment (Figure 5(a)). However, in these cells, caspase-3 was already active after 30 min as demonstrated by z-VAD treatment (Figure 5(b)). Staurosporine was activating caspase-3 and caspase-7 after 30 min and even more after 24 h compared to the untreated cells. However, statistical significance was not reached comparing control treatment to staurosporine treatment, even if a tendency towards significance increased at 24 h (*P* = 0.08) compared to 30 min (*P* = 0.92). After 30 min, caspases were already activated but cell death was not observed, whereas after 24 h cells were dead and caspases could not be activated further. A comet assay carried out under the same conditions (Figure 5(c)) revealed that the mean comet tail length was not diminished by z-VAD in presence of A*β* after 30 min compared to the A*β* treatment. In this case, DSBs were not due to activated caspase-3. After 24 h, the mean comet tail length was significantly reduced by z-VAD in presence of A*β* suggesting the production of DSBs due to activated caspase-3. Overall, A*β*-induced DSBs appear not to be related to proapoptotic events after 30 min treatment.

Furthermore, cell viability was measured by quantification of intracellular ATP in comparison to cells lysed by digitonin. After 30 min A*β* treatment in presence or not of RA, DI TNC_1_ cell viability was neither decreased nor increased, whereas it was the case subsequent to the digitonin treatment (Figure 5(d)). An identical observation was made for SH-SY5H cells (not shown). Cells from 4-month-young neocortical tissue showed after 1 h a much higher viability than cells from 16-month-old tissue (Figure 5(e)), suggesting a higher fragility of the latter tissue in the* in vitro* conditions. Overall viability dropped after 30 min A*β* treatment due to decreased intracellular ATP in the young tissue (*P* = 0.001). However, this decrease was less significant when RA was added to A*β* for both young and old cortical tissues (*P* = 0.036). We observed an increase in viability of about 53% for the young tissue and of about 59% for the old tissue, indicating that under the experimental conditions RA enhances viability at a time when DSBs are being repaired.

## 4. Discussion

Our main observation is that RA is involved in A*β*-induced DSBs repair in neuronal and astrocytic cell lines as well as in the cortical tissue, likely in cortical neurons as well as astrocytes, and in young and even in aged mice. Truly the addition of RA after DSBs production by A*β*_1–42_ treatment can repair DSBs. This was shown by two independent methods: comet assay and gel electrophoresis with SH-SY5Y neuroblastoma cells. Thus, according to these data, RA and its receptor (RAR) are involved not only in the prevention of A*β* synthesis, A*β* oligomerisation, and plaques removal, as already shown [29, 32–34, 36], but also in the A*β*-induced DSBs repair.

### 4.1. A*β*_1–42_ Induces DSBs with a Pathological Potential

We choose to grow SH-SY5Y and DI TNC_1_ cells in presence of FCS 1% instead of FCS 10%. In both cases, A*β* induced significantly more DSBs, but in the former condition cells were more differentiated as in the cortical tissue. L-Glutamine was also added as it is already known to protect against DSBs formation [16] and only the effect of RA had to be tested. L-Glutamine was equally added to the homogenized cortical tissue but B27 supplement was avoided as it contains RA. Interesting was the observation that only the most pathological form of A*β*, A*β*_1–42_, was inducing DSBs in both of the SH-SY5Y and DI TNC_1_ cells, suggesting that A*β*_1–42_-induced DSBs might play a role in Alzheimer's pathology. Noteworthy is also the fact that A*β*_1–42_ treatment appeared to be less efficient in inducing DSBs in the aged than in the young murine cortical tissue. This cannot be due to the state of the A*β* that corresponds to a mixture of monomer and oligomers in both cases [44] but rather to the state of the tissue, such as a lower metabolism of the aged cortical tissue.

### 4.2. RA Has a Counteracting Effect on DSBs Production

RA has been shown to reduce DSBs in a concentration-dependent manner in SH-SY5Y cells. 5 *μ*M RA appears to be optimal for decreasing DSBs and increasing cell viability, that is, ATP concentration, compared to the untreated cells. Higher or lower RA concentrations resulted in unchanged DSBs levels compared to the control cells, whereas increased RA concentrations only decreased cell viability. This variation of RA effect according to its concentration is compatible with the observation that when neuronal activity is low, endogenous RA synthesis is locally activated in order to maintain the structures of neurons (homeostatic synaptic plasticity) and that conversely RA synthesis decreases when neuronal activity increases [20]. It has to be observed that the DSBs levels following the RA treatments were not consistently lower than the background DSBs levels of the untreated samples. This phenomenon might be due to variations of endogenous RA levels in relation to cell differentiation or division. Furthermore, RA was shown to decrease A*β*-induced DSBs through the RAR*α*/*β* receptors by using the Am80 agonist and the AGN 193109 antagonist. The agonist being as effective as RA in reducing DSBs, an effect of RA through another receptor, for example, RXR-PPAR*β*/*δ* [42], appears excluded in SH-SY5Y cells. The observation that the HDAC inhibitor TSA, which enables derepression of Retinoic Acid Response Element-dependent gene expression, results in a similar decrease of A*β*-induced mean comet tail length as RA strengthens the role of RAR in repair of A*β*-induced DSBs. Reparation might occur either through expression of genes involved in DSBs repair or more directly through chromatin decondensation at sites of RA-dependent gene expression allowing access to proteins involved in DSBs repair. One of these proteins is DNA-PK of the NHEJ repair pathway. However, its inhibitor NU 7026, used at a concentration of 150 *μ*M, resulted only in a partial repair of A*β*-induced DSBs compared to the RA treatment. A concentration effect of NU was excluded as a dose-response curve demonstrated that mean comet tails lengths reached a maximum size at 150 to 250 *μ*M NU and decreased significantly at 50 to 100 *μ*M NU (data not shown). The ATM inhibitor, KU 55933, resulted equally in a partial repair of A*β*-induced DSBs. We showed in SH-SY5Y cells that both of DNA-PK and ATM, known to be complementary [45, 46], enable the complete counteracting effect of RA on DSBs production.

### 4.3. Adaptive Cellular Response due to RA-Mediated DSBs Repair: The DSB-PRAE Hypothesis

We observed that short-term (not long-term) treatment with A*β* is not decreasing cell viability or causing cell death and that caspase-3 was activated at the same level in presence of A*β* or not in SH-SY5Y grown with 1% FCS. Indeed, even staurosporine is known to induce neurite outgrowth in the short term before inducing cell death and apoptotic DNA fragments in the long term [47]. We also did not observe apoptotic fragments of *n*  × 180 bp after 30 min A*β* treatment, suggesting that apoptosis is not yet activated under these conditions. However, at that time, DSBs are induced by A*β*, and if not repaired in the longer term, they might be deleterious [48]. Furthermore, in the short time, RA can repair DSBs and appears even to increase viability in the cortex of young and to a lesser extent of aged mice. The fact that RA is repairing DSBs as Am80 and trichostatin A and that both of them alter memorization [49, 50] suggests a similar role for RA. We make the hypothesis that whereas A*β* produces DSBs, RA results in targeted error-prone repair of the subset of DNA regions with RA-dependent gene expression, involved in particular in neuronal or synaptic maintenance [23]. This process of selective DNA repair might result in a neuronal state adapted to the metabolic changes occurring with aging. This phenomenon of “DSBs production and repair-dependent adaptive gene expression” (DSB-PRAE hypothesis) would remain functional up to a pathological threshold reached by A*β* increase and RA decreased response with aging and might be an explanation for the role of DSBs in memorization [7].

In conclusion, we have found that RA repairs A*β*-induced DSBs in neural cells of the cerebral cortex. Our data are in favor of a role of RA in increasing neural viability and also possibly in providing a neuronal adaptive response. However, this should be demonstrated notably in an AD mouse model. Furthermore, our data suggest that a better knowledge of the mechanisms involved in A*β*-induced DSBs might provide additional means to target pathological A*β*-induced changes, not just by impairing the A*β*-amyloid cascade but by repairing some of its consequences.
